# Hope Walks: The Impact of Clubfoot Treatment on Human Flourishing in Ethiopia

**DOI:** 10.1002/hec.70040

**Published:** 2025-09-18

**Authors:** Bruce Wydick, Gianna Camacho, Patrizio Piraino

**Affiliations:** ^1^ University of San Francisco San Francisco California USA; ^2^ University of Notre Dame Kellogg Institute for International Studies and CEIDS Notre Dame Indiana USA; ^3^ Department of Agricultural and Resource Economics University of California at Davis Davis California USA; ^4^ Department of Public Health Commonwealth of the Northern Mariana Islands Saipan Northern Mariana Islands

**Keywords:** clubfoot, Ethiopia, human flourishing, pediatric surgery

## Abstract

Children born with severe congenital conditions in low‐income countries rank among the most disadvantaged among the global ultra‐poor. We study the impact of clubfoot and its treatment across multiple dimensions of human flourishing on data collected from 564 children in Ethiopia. Working with Hope Walks, an organization that funds clubfoot interventions in numerous countries, we use a quasi difference‐in‐differences approach that generates counterfactual outcomes from the nearest‐age siblings of children born with clubfoot, nested within a family‐level fixed effect. We find that clubfoot status (early treatment) results in an impairment (restoration) of −1.44σ (0.91σ) in physical mobility, −1.17σ (0.79σ) in mental health, −1.07σ (0.64σ) in social inclusion, −0.48σ (0.98σ) in an education index, −0.76σ (0.42σ) in religious faith, and −1.32σ (0.94σ) in an aggregate index of human flourishing (all p<0.05). We attribute the large, broad, and significant impacts from clubfoot treatment to (i) a highly effective medical intervention that is (ii) carried out in an impoverished setting with scarce existing support for children born with disabilities, which (iii) broadly generates spillover effects across key development outcomes.

## 1 Introduction

The World Health Organization (2023b) estimates that approximately 1.3 billion people worldwide experience a significant disability, representing 16% of the global population. Due to higher congenital risk factors, disability is more prevalent among the global poor. According to the World Health Organization (2023a), nine out of 10 children born with a congenital disorder are in low‐ and middle‐income countries (LMICs). In low‐income settings, children with serious congenital conditions face some of the most severe challenges, particularly among the ultra‐poor. This study contributes to the growing literature on the economics of disability in LMICs, which has expanded since the 2008 United Nations Convention on the Rights of Persons with Disabilities (CRPD) and the 2030 Sustainable Development Goals (SDGs), both of which emphasized improving the welfare of individuals with congenital disabilities in these settings. We employ quasi‐experimental methods to estimate the impact of congenital clubfoot and its treatment on an array of life outcomes for Ethiopian children.

Prior reviews of the economics of disability in high‐GDP countries (Strauss and Thomas 1998; Currie and Madrian 1999) have focused on the negative effects of disability on labor market outcomes. More recently, Mitra and Palmer (2023) have reviewed the literature on the complex economic relationship between disability in LMICs and global poverty, particularly in the context of the CRPD and emerging global efforts to address disability in these settings. A growing body of evidence demonstrates that individuals with disabilities face greater deprivations than their non‐disabled peers across various dimensions, including poverty, education, employment, and health (Mitra and Palmer 2023). While previous research has explored the causal effects of disability on well‐being (e.g., Takasaki 2020), less attention has been paid to how early life disadvantages contribute to long‐term disability‐related deprivations. Furthermore, there is a limited amount of research that utilizes quasi‐experimental methods to assess the impacts of congenital conditions on developmental outcomes and the effectiveness of health interventions aimed at mitigating these negative effects. Our research aims to fill this important gap.

Approximately 1 in 1000 children globally are born with *talipes equinovarus*, commonly known as clubfoot, a rate similar to births with cleft lip or palate. While readily and effectively treated in high‐income countries, clubfoot often goes untreated in LMICs. Untreated congenital conditions like clubfoot can lead to disabilities that may increase poverty rates in LMICs through indirect impacts on social inclusion, education, employment, and earnings (Mitra et al. 2013). To our knowledge, no prior research has rigorously employed quasi‐experimental methods to examine the effects of congenital clubfoot and its treatment on children's life outcomes. Although the medical literature on clubfoot is extensive in many areas, it does not aim to estimate the causal effects on life outcomes of either congenital clubfoot status or receiving treatment. While the exact determinants of clubfoot are still under medical investigation (Hegazy et al. 2020), its occurrence is likely correlated with family genetics, environmental factors, and maternal behaviors such as smoking (Honein et al. 2000). Consequently, simply comparing outcomes of children with clubfoot to the general population is unlikely to yield valid counterfactuals for either the condition itself or its treatment.

In partnership with Hope Walks, a faith‐based development NGO facilitating clubfoot treatment in 14 countries, we study the causal effects of congenital clubfoot status and its treatment on an array of holistic life outcomes. We generate a novel dataset from interviewing mothers of 564 Ethiopian children, compiling information on children born with clubfoot and their nearest‐age siblings. In our sample, 59.9% of children born with clubfoot were treated and 62.9% of the treated children received early treatment which, according to the existing medical literature, maximizes treatment effectiveness. Through incorporating a mother‐level fixed effect and including binary independent variables for clubfoot status, treatment, and early treatment, we are able to estimate (i) the loss in standard deviations over five facets of human flourishing from congenital clubfoot status; (ii) the degree to which the clubfoot intervention restores these outcomes; and (iii) the added restoration benefits from early treatment which, based on best practices, is defined here as occurring before 6 months of age.

We estimate average treatment effects on the treated (ATT) across standardized indices of physical, psychological, social, educational, and faith outcomes, all outcomes of interest to our partner NGO.^1^ First, our results reveal an immense threat to human flourishing, as based on these outcomes, among children born with clubfoot. We find stark reductions of 1.44σ in physical mobility, 1.17σ in mental health, 1.07σ in social inclusion, 0.48σ in an education index, 0.76σ in the faith of their religious community, and 1.32σ in an aggregate index of human flourishing (all significant at p<0.001).

Second, we show that early treatment of clubfoot restores outcomes in each of these areas significantly closer to, but not generally equal to, those of a child's nearest‐age sibling. We find that early treatment leads to a restoration of 0.91σ in physical mobility, 0.79σ in mental health, 0.64σ in social inclusion, 0.98σ in our education index (where the intervention actually yields restoration that exceeds the negative impact of clubfoot), 0.42σ in religious faith, and 0.94σ in our aggregate index of human flourishing (all significant at p<0.05).

Our results contribute to both the research literature and to health practices in LMICs. First, we quantify the severe impacts stemming from a common congenital condition on the everyday lives and functioning of children in low‐income countries, where aside from direct effects on physical mobility, clubfoot causes more than a standard deviation loss in both mental health and social inclusion.

Our second key contribution is in showing that the effects of clubfoot treatment initiated past infancy are not significantly different from zero. This holds true across all five of our measured dimensions of human flourishing, and strongly supports existing medical guidelines that recommend commencing treatment of clubfoot in the weeks shortly after a child is born and diagnosed.

Finally, we find that the ATT of early clubfoot intervention on a broad array of development indicators is extremely high relative to the impacts of most educational and health interventions in LMICs. For example, the estimated effects of early clubfoot treatment are both significantly higher and more precisely measured than the impacts of cleft palate surgeries carried out by Operation Smile (Wydick et al. 2022). We attribute these unusually high ATTs to both the efficacy of early‐age intervention and the severe counterfactual outcomes faced by individuals with untreated congenital conditions in LMICs. Based on substantial welfare gains in the lives of treated children, our results suggest a significant reallocation of resources directed to early treatment of congenital impairments in LMICs more generally and to clubfoot treatment in particular.

In the next section, we provide background on the clubfoot condition and its treatment, as well on the origin of our data. Section 3 presents our model and ATT estimates from the Hope Walks intervention and discusses barriers to treatment as revealed in our Ethiopia survey. Section 4 reflects on our results and discusses program and policy implications.

## 2 Background and Data

### 2.1 Effects of Disabilities in LMICs

The social and economic costs of disabilities in LMICs are substantial. Filmer (2008) finds a 10‐percentage‐point increase in the probability of falling into the two poorest quintiles of poverty in LMICs for disabled individuals, which the authors ascribe to a lack of opportunity to engage in the local economy. Disability is also found to be significantly associated with higher rates of multidimensional poverty due to low education and skill accumulation, leading to significantly reduced earnings in adulthood (Mitra et al. 2013).

*Talipes equinovarus*, commonly referred to as clubfoot, is an inborn three‐dimensional deformity of the leg, ankle, and foot. Globally, it is one of the most common congenital impairments in newborns with about 80% of clubfoot cases occurring in LMICs (Gupta et al. 2006). Medical professionals familiar with clubfoot advise seeking treatment as early in an infant's life as possible. In high‐income countries, deformities associated with clubfoot are often recognized quickly after birth, or *in utero* through ultrasound scans; treatment is widely accessible and can be carried out with no major delays. Unfortunately, this is not the case in many LMICs, leading to numerous individuals living entire lives with the discomfort and restrictiveness of untreated clubfoot. Basit and Khoshhal (2017) present evidence that uncorrected structural defects of the foot and lower leg tissues can cause abnormal positioning of the foot and ankle joints. This abnormal positioning typically results in malformation of joints and ligaments, severe discomfort, and long‐lasting disability if left untreated.

Deformities associated with clubfoot can be characterized into four components: equinus at the ankle, varus at the hindfoot, forefoot adductus, and cavus (Gupta et al. 2006). All four components of clubfoot can be measured or “scored” using the Pirani Scale, a tool that assesses the severity of each of the components of clubfoot.^2^ Generally, the goal of treatment for any form of clubfoot is to attain a functional, pain‐free, plantigrade foot with good mobility (Gupta et al. 2006). Studies focused on the efficacy of alternative clubfoot treatment methods have shown that a minimally invasive and economical treatment plan, the Ponseti Method, has proved successful in achieving treatment goals (Bor et al. 2009).

Hope Walks utilizes the Ponseti method in all of its work in LMICs, where the medical intervention costs approximately US$500 per patient. The Ponseti method uses several plaster casts, often combined with an Achilles tenotomy, followed by a period of nightly bracing until the age of four to maintain the foot in the corrected position (Tuinsma et al. 2018). Because it relies on bone growth to correct the effects of clubfoot, the orthopedic community consensus is that the Ponseti method is most effective when initiated as early in life as possible. It is recommended that newborns with signs of clubfoot may be referred to a clubfoot center preferably within 48 h but no more than 1 week following delivery (Besselaar et al. 2017). However, seeking treatment within this time frame is often impossible in LMIC contexts.

Results from Bor et al. (2009) provide evidence of the physiological success of the method, where 89.2% of feet in their sample achieved at least a “good” outcome. Ippolito et al. (2003) present a comparison between babies treated with the Ponseti Method relative to an alternative Marino‐Zuco method. They show a 78% success rate in the Ponseti Method compared to a 43% success rate in the Marino‐Zuco group. In addition, the Ponseti method has lower costs, increased accessibility, and overall treatment efficiency; all of which make it particularly suited for implementation in LMICs, where there are fewer orthopedic surgeons or specialists (Gupta et al. 2006).

The existing literature on clubfoot focuses primarily on the success of the procedure from a medical/physiological perspective. Studies in this literature, however, generally fail to establish valid controls and/or counterfactuals, thus falling short of establishing a basis for understanding the causal effects of congenital clubfoot status and clubfoot interventions. In addition, most studies report medical results from treatment, with limited evidence on both the impact of clubfoot on life outcomes more generally, and on the degree and nature of restoration of these life outcomes with treatment. Our study is both the first quasi‐experimental study on clubfoot intervention and the first to estimate effects across a broad array of child's life outcomes. The outcomes we study also serve as an evaluation of the mission objectives of Hope Walks as a non‐profit organization. Hope Walks is an NGO whose stated mission extends beyond clubfoot repair to a broader conception of multi‐dimensional human flourishing nested in the general framework of “integral human development” common to the faith‐based NGO community today.

### 2.2 Data

We interviewed parents through Cure International hospitals in the Addis Ababa region and the surrounding areas of Adama and Hawassa. We designed surveys to be carried out via phone interviews due to the COVID‐19 pandemic. All of the 564 children about whom we collected data were either past patients or were on the waitlist for treatment with Hope Walks from these partner hospitals. The first wave of data was collected between November 2021 to March 2022, while the second wave was collected from September to December 2023.

There are two distinct sample groups within this study. The first group includes treated patients and their nearest‐age sibling. To be included in the treated group, children must have been between 6 and 18 years of age and born with congenital clubfoot. Additionally, they should have been fully treated or at least in the final stages of casting in the Ponseti intervention to be considered “treated”.

The second group that was examined in our sample were yet untreated patients and their respective nearest‐age siblings. Individuals in this group must have been between the ages of 6 and 18 years old and born with congenital clubfoot, and had not yet begun treatment but were scheduled to begin treatment with one of the associated organizations. To find these individuals, we used a roster of patients who were scheduled to start treatment within the upcoming calendar year. The information on patients and their siblings, for both treatment groups, was obtained over the phone, at the same time, in the same location, and using the same survey to ensure that there were no confounding factors influencing the responses.

Our survey consisted of two main sections, the first of which aimed to develop a respondent profile by asking about basic demographic data as well as questions about the treatment plan and physical health of the sibling born with clubfoot. In this section, we record their contact information, the number of children in the family unit, languages spoken, and any religious affiliations. Additionally, the survey respondent was also asked about the child's initial diagnosis, such as the age at diagnosis and initial Pirani score. To conclude this section, we noted their current stage of treatment.

The second section of the survey focused solely on life outcomes. The first data gathered within this section relate to physical and mobility capabilities. We asked parents to rate their child with clubfoot and their nearest age sibling on a formal six‐point mobility scale used commonly to assess clubfoot severity. Other questions in this domain include how easily their children can complete everyday activities, such as walking or partaking in sports. The following sub‐section focused on psychological and faith questions. Parents were asked about the hopes and aspirations of their children. We also asked about the prevalence of anxiety, depression, and nervousness in children, as well as happiness, and involvement with their family's religious community. The last two domains analyzed within this section relate to social and educational outcomes. Parents were asked about the social behavior of their children and about the degree of social inclusion their children have within the community. We then collected information on children's educational attainment–i.e., if currently enrolled in school, when and why they dropped out of school (if applicable), and academic performance relative to other children their age.

## 3 Empirical Model and Results

### 3.1 Theory of Causal Change

**FIGURE 1:**
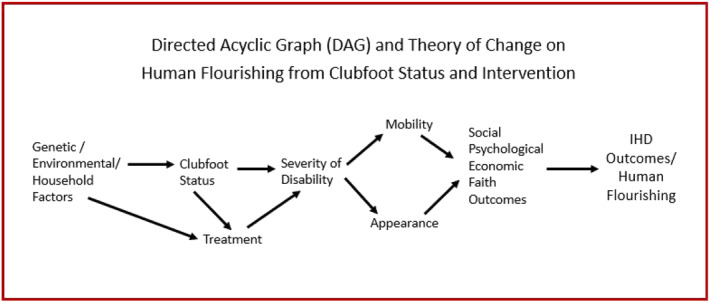
DAG: How clubfoot status and treatment affect IHD outcomes.

A Directed Acyclic Graph (DAG) illustrating our empirical research framework and theory of change is given in Figure 1. The current body of medical knowledge attributes clubfoot to genetic, and possibly environmental and parental characteristics, such as maternal smoking and nutrition levels (Dobbs and Gurnett 2009). Household characteristics may also lead to treatment (and the timing of treatment), which then affects the severity of the disability. The severity of the clubfoot (mitigated by treatment and proper timing of treatment) affects the appearance of a child's feet and legs and a child's mobility, which then jointly affect social, psychological, economic (including education) and faith variables, all of which relate to human flourishing in the integral human development framework adopted by Hope Walks.

To identify the effects of clubfoot status and clubfoot treatment, it is necessary to account for the backdoor path to the severity of disability through genetic, environmental, and household factors. This can be done through a household‐level fixed effect if we assume that siblings share common genetic makeup, maternal behaviors and environmental exposure.

### 3.2 Empirical Model

We analyze the causal effects of clubfoot status and its treatment on five facets of human flourishing using a cross‐sectional difference‐in‐differences method nested within a household fixed effect. Our estimation compares child life outcomes with treated clubfoot to the life outcomes of their nearest‐age sibling. This difference is then compared to the difference between the life outcomes of untreated clubfoot patients and their own nearest‐age siblings.

This identification framework requires three assumptions for obtaining unbiased ATT estimates of the intervention. The first assumption is that the occurrence of clubfoot occurs randomly to a sibling *i* within a given household *j*. Second, any factors (positive or negative) that affect both a child's selection into clubfoot treatment and life outcomes are common to all siblings. Third, the potential outcomes for clubfoot patients and siblings are constant and independent of clubfoot or treatment status–i.e., the stable‐unit‐treatment‐value (SUTVA) assumption. Below, we discuss implications for our estimates from violations of these assumptions.

We estimate the following OLS equation using family‐level fixed effects:

(1) $$Y_{ij}=\alpha +\beta B_{i}+\tau_{1}T_{i}+\tau_{2}ET_{i}+X_{i}^{\prime }\gamma +\theta_{j}+\epsilon_{ij}$$

where $Y_{i_{j}}$ represents outcomes that include physical/mobility, psychological health, social inclusion, education, and religious faith. $T_{i}$ is a binary variable indicating clubfoot treatment, $ET_{i}$ is a binary variable for early treatment (commencement of treatment before 6 months of age), $X_{i}$ is a vector of controls that include age, birth order, and gender, $\theta_{j}$ is a family‐level fixed effect, and $\epsilon_{i_{j}}$ is the error term. The effect of early treatment is $\tau_{1}+\tau_{2}$.

### 3.3 Threats to Identification

The main threats to the identification of average treatment effects on the treated in our estimates are (i) non‐random instances of clubfoot across our sample population that are correlated with life outcomes; and (ii) violations of the SUTVA assumption, where instances of clubfoot, its treatment, and/or the age at treatment depend on the treatment status of others in the sample.

With respect to the first issue, both the genetic and environmental factors that are believed to cause clubfoot (Dobbs and Gurnett 2009) should be common among siblings. It is about twice as prevalent among boys (McConnell et al. 2016), true in our sample as well, with slightly more prevalence in younger siblings in our data (average birth order 0.21 higher), where we control for both of these attributes. Conditional on these attributes and a mother‐level fixed effect, both medical evidence as well as our own data and suggest clubfoot then manifests randomly across siblings.

With respect to spillovers, the counterfactual generated in our estimation is the outcome of the nearest‐age sibling of a child born with clubfoot, where the nearest‐age sibling could be either younger or older. Unbiased estimation thus requires satisfying the SUTVA assumption in which clubfoot status of one sibling does not impose externalities on the outcomes of nearest‐age siblings.

While constructing an argument for *positive* spillovers from congenital clubfoot status to a nearest‐age sibling is difficult, such externalities could arise through a re‐allocation of parental investment in children (see Currie and Almond 2011; Jayachandran and Pande 2017). One child's disability could influence resource allocation, leading to positive effects on educational and other outcomes if parents reallocate schooling resources away from the child with a disability toward their other children. Such positive spillovers would generate an upward bias for both the estimated disadvantage of congenital clubfoot status and the restorative effect of treatment.

We view it more likely that the SUTVA assumption could be violated by *negative* spillovers from a child's disability onto other siblings. This may occur especially if non‐impaired siblings are expected to help provide care for a disabled brother or sister (Joseph et al. 2023). It is also conceivable that a child's own mental health and/or social inclusion could be negatively affected by their sibling's clubfoot status. In general one should view our estimates as being inclusive of average parental and sibling responses to the treated or untreated clubfoot status of a sibling.^3^ If negative spillovers exist onto siblings from children born with clubfoot, this would also create a positive spillover from treating clubfoot. These effects would tend to bias both the impacts from congenital clubfoot and its restoration downwards. We test for the presence of these externalities by removing the household level fixed effect and testing for differences in outcomes between siblings of treated and untreated children born with clubfoot.

### 3.4 Descriptive Statistics

Table 1 gives summary statistics for our sample of Ethiopian children. Our sample consists of pairs of siblings, one born with clubfoot and this child's nearest age sibling. Table 1 column (1) gives the mean values for children whose sibling is an untreated child with clubfoot. The mean outcomes for the untreated sibling are displayed in column (2). Column (3) shows the outcomes for the sibling of a treated child, who can then be compared to their treated sibling in column (4). The final columns contain *p*‐values from *t*‐tests between means in columns 1 and 2 and between means in columns 3 and 4. The table shows that families of untreated children tend to be poorer and more rural than those with treated children, differences we control for through the household‐level fixed effect.

**TABLE 1:** Summary statistics: Hope walks.

	(1)		(2)		(3)		(4)		(5)	
	Sib, Untreated		Untreated CF		Sib, Treated		Treated CF		*p*‐values	
	Mean	SD	Mean	SD	Mean	SD	Mean	SD	1–2	3–4
Age of child	8.95	4.75	11.40	3.25	10.32	4.68	7.92	2.03	0.00	0.00
Male gender	0.62	0.49	0.62	0.49	0.56	0.50	0.66	0.48	0.94	0.06
Birth order	2.67	1.66	2.83	1.86	1.82	1.08	2.10	1.25	0.50	0.03
Monthly Inc (Birr)	3142	2407	3223	2404	5905	11,215	5750	10,712	0.82	0.99
Family in Agric	0.80	0.40	0.80	0.41	0.62	0.49	0.62	0.49	0.97	0.98
Mobility index	0.61	0.36	−0.80	0.89	0.61	0.23	−0.43	1.19	0.00	0.00
Psych health index	0.51	0.31	−0.65	1.07	0.43	0.39	−0.30	1.25	0.00	0.00
Aggr social index	0.47	0.61	−0.61	1.07	0.39	0.64	−0.26	1.13	0.00	0.00
Educ index	0.19	0.89	−0.09	1.03	0.20	0.81	−0.25	1.14	0.03	0.00
Faith index	0.47	0.71	−0.27	1.15	0.21	0.80	−0.31	1.06	0.00	0.00
IHD index	0.59	0.42	−0.68	0.98	0.50	0.39	−0.39	1.20	0.00	0.00
Observations	112		116		163		173	112	163	

*Note: p*‐values test differences between means in columns 1 and 2 and differences in means in columns 3 and 4, respectively.

### 3.5 Impacts on Human Flourishing Outcomes

Here we estimate the impact of both clubfoot status and clubfoot treatment on physical mobility, psychological outcomes, social inclusion, education, and religious faith. Each of these are created from an index of individual outcomes from our survey data. A more detailed analysis of the impacts of clubfoot status and treatment within each of these areas can be found in the Appendix. From these five main outcome areas we create an index of overall human flourishing using the method of Kling et al. (2007), which consists of the standardized sum of each of these standardized indices.

As seen in Table 2, clubfoot status causes an enormous reduction in physical mobility of 1.44σ (*p* < 0.001). Physical mobility (col. 1) is comprised of an index of the distance a child is able to walk, comfort in walking, ability to participate in and enjoy sports, and frequency of reports of tired legs and feet. Early treatment does not fully restore physical mobility, but does so substantially by 0.91σ (*p* < 0.001). Table 2 also shows that treatment initiated past 6‐months of age does not display any statistically significant impact on restoration of physical mobility. Estimates for treatment effects on individual outcome variables comprising these indices are provided in the Supporting Information S1.

**TABLE 2:** Summary outcomes: Human flourishing.

	(1)	(2)	(3)	(4)	(5)	(6)
	Physical	Psych	Social	Educ	Faith	HF index
Born clubfoot	−1.443***	−1.171***	−1.071***	−0.477***	−0.762***	−1.320***
	(0.0893)	(0.0922)	(0.0892)	(0.116)	(0.0942)	(0.0836)
Treated clubfoot	−0.188	−0.117	−0.0368	−0.420	−0.0721	−0.183
	(0.196)	(0.176)	(0.165)	(0.238)	(0.156)	(0.180)
Early treat	0.905***	0.790***	0.642***	0.980***	0.415*	0.937***
	(0.212)	(0.167)	(0.172)	(0.249)	(0.160)	(0.189)
Born clubfoot	−1.413***	−1.145***	−1.050***	−0.445***	−0.748***	−1.29***
	(0.0886)	(0.0914)	(0.0888)	(0.115)	(0.0937)	(0.0822)
Treated clubfoot	0.310*	0.317*	0.316**	0.120	0.156	0.332**
	(0.135)	(0.129)	(0.120)	(0.167)	(0.120)	(0.124)
*N*	564	564	564	564	564	564
MotherFE	Yes	Yes	Yes	Yes	Yes	Yes

*Note:* Upper regression includes control for early treatment; lower regression does not control for early treatment. Standard errors clustered at the household level in parentheses. Regressions control for age, gender, and birth order of children. Percent restoration of human flourishing index with early surgery = 71.0%. Joint (Index) Test of Clubfoot + Treatment + Early Treatment, −0.57, *p* < 0.01. Joint (Index) Test of Clubfoot + Treatment, −0.96, *p* < 0.01.

* *p* < 0.05.

** *p* < 0.01.

*** *p* < 0.001.

Clubfoot status also causes starkly lower levels of mental health, reducing a psychological health index based on sub‐indices of parental reports of self‐esteem, aspirations, anxiety, and depression by 1.17σ (*p* < 0.001). Again, early treatment is essential and restores mental health by 0.79σ (*p* < 0.001), or about 68% of the decline in mental health caused by clubfoot birth status.

Table 2 shows the dramatically lower levels of social inclusion faced by children born with clubfoot in our Ethiopian sample. Untreated clubfoot causes a 1.07σ (*p* < 0.001) reduction in social inclusion, which is an index created from questions related to the number of other children a child would call a friend, frequency of leaving the home to be with friends, inclusion in social circles, whether a family is “proud to have the child in the family”, and frequency of bullying. Early clubfoot treatment restores about 60% of this social inclusion index, with a positive impact of 0.64σ (*p* < 0.001).

We find somewhat smaller (but still precisely measured) impacts from clubfoot birth status on education. Our index of educational outcomes consists of whether a child attended any school before the first year of primary school (pre‐school or kindergarten), current enrollment status, current grade level, and academic performance. Our estimates show a 0.48σ loss in our education index from clubfoot status but a 0.98σ (both *p* < 0.001) gain from early clubfoot treatment, more than closing the gap with the education outcomes of nearest‐age siblings. We speculate that this may be related to children with remaining physical challenges finding a comparative advantage in education and vocations for which physical labor is less essential.

Hope Walks is a faith‐based NGO with missional objectives related to a conception of human flourishing that includes faith outcomes. An important question for the organization is the extent to which being born with a disability such as clubfoot prevents a person from participating in their religious community or perhaps even causes them to question the values, faith, or religious beliefs of their family and community. Religious belief and activity plays a central role in Ethiopian culture, and participants in our study were from three religious groups: Muslim (27.1%), Orthodox Christian (47.9%), and Protestant Christian (25.0%). Our survey questions were general and were intended to be applicable to all of these groups. Our faith index is comprised of questions that asked about the degree of involvement of a child in their local faith community, the importance of religious belief in the child's life, and whether the child participates in religious youth activities. Clubfoot status causes a decline in this index of 0.76σ.^4^ Early clubfoot treatment restores most of this negative impact (0.42σ, *p* < 0.05), but less fully (55.2%) than other outcome areas.

Clubfoot status among children reduces our aggregate human flourishing index by 1.32σ (*p* < 0.001); early treatment restores the index by 0.94σ (*p* < 0.001), a restoration of human flourishing of 71.0%. Again we note that all significant impacts that we see across areas of human flourishing, including the aggregated index, are driven by early treatment.

**FIGURE 2:**
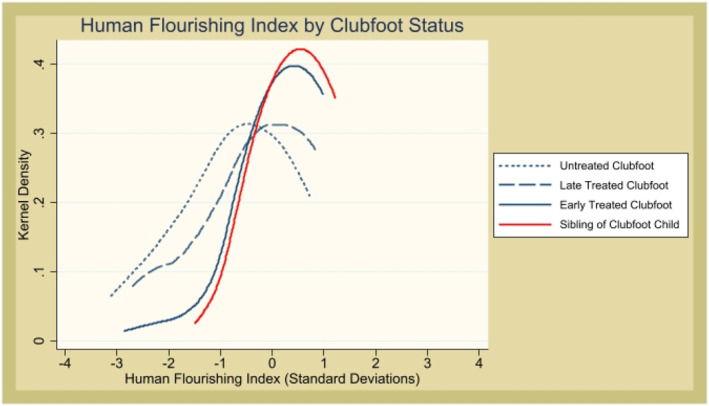
Human flourishing index across clubfoot treatment status.

In the lower panel of Table 2, we show estimations that combine treatment at all stages, where the coefficients on treated clubfoot retain precision but are now substantially lower, as a result of including children treated after 6 months of age. Figure 2 shows kernel density functions of our human flourishing index across treatment status.

### 3.6 Robustness Checks

#### 3.6.1 Alternative Indices

In our main estimations, we use a standard Kling et al. (2007) indexing procedure, but as a robustness check, we regenerated each of our mobility, psychological health, social inclusion, faith, and education indices using the method of Anderson (2008). This method corrects for covariance between components, weighting down variables that are highly correlated with others in the index, and weighting up variables that show higher unique variation. As seen in Supporting Information S1: Table A11 none of our results or levels of significance substantially change for any of these indices. Our human flourishing index gives somewhat smaller point estimates, especially on the impact of clubfoot status, 1.06σ (*p* < 0.001). The impact of early treatment is 0.88σ (*p* < 0.001) and the restoration percentage increases to 81.5%. We also carried out robustness checks using alternative sets of control variables, trimming our sample to closer age bands, and running estimations conditional on different levels of income. In each of these checks, we find strongly congruent impacts on our outcome indices from clubfoot status and treatment, with similarly high levels of significance.

#### 3.6.2 Check for Externalities

We also examine potential externalities on the siblings of children born with clubfoot. If negative externalities exist, we would expect better outcomes among siblings of treated children, while the opposite would suggest positive externalities. To test this, we run regressions on nearest‐age siblings, excluding household fixed effects while controlling for household characteristics such as income, employment, urban/rural and agricultural status, religion, and number of siblings. Overall, we find almost no statistically significant differences in outcomes between nearest‐age siblings of treated and untreated children with clubfoot.

The only exception arises when we define treatment strictly as early treatment. In this case, we find modest evidence of higher educational outcomes among siblings of untreated children, suggesting mild support for the idea that parents may reallocate educational resources away from the disabled child. However, we find no similar effects in other outcome areas, leading us to conclude that any widening or narrowing of outcome gaps between siblings is essentially driven by the presence of congenital clubfoot and the effects of early‐age treatment.

#### 3.6.3 Restrictions on Nearest‐Age Siblings

We conduct an additional robustness check on externalities by limiting our estimations to cases where the nearest‐age sibling is older than the child born with clubfoot. Among the 288 children in these sibling pairs, we find that the impact of early treatment remains statistically significant at *p* < 0.01 across all cases, with the effect on human flourishing nearly unchanged.

When we further restrict the sample to the 276 children whose counterfactual outcomes are based on younger siblings (or twins), all outcome areas retain statistical significance, except for faith and spirituality. Notably, the impact on the educational index doubles to 1.20σ, while the effect on overall human flourishing remains consistent.

#### 3.6.4 Impacts Driven by Single Variables Within an Index

It is possible that the outcomes we report in our indices are driven by just one or two key variables. To examine this, we provide a detailed breakdown in the Supporting Information S1, showing the impacts of congenital clubfoot status and treatment on each variable within our indices. As shown in the appendix tables, all 10 component outcomes for physical well‐being are significantly affected by clubfoot status, and 8 show significant improvement with early treatment (Supporting Information S1: Tables A1 and A2). In the psychology index, all 18 measures—covering self‐esteem, hope, aspirations, anxiety, and depression—are negatively impacted by clubfoot status, with 13 significantly improving after early treatment (Supporting Information S1: Tables A3–A6). For social inclusion and behavior, 10 of 12 outcomes are negatively affected, and 6 show significant improvement with early treatment. In education, two of four outcomes are significantly negatively impacted, while three of four improve significantly with early treatment. Finally, for faith and spirituality, all three outcomes are affected by clubfoot status and significantly improve with treatment. Notably, a child's belief that “faith is important to them” is restored even with late treatment. In summary, the improvements in our indices are not driven by just one or two outcomes but rather by the majority of components within each index.

#### 3.6.5 Subjective Parental Measurement

Our data in this study are taken from parents, typically the mother of the child born with clubfoot and their nearest‐age sibling. One concern with some of our more subjective outcomes, for example, psychological wellbeing and impacts on religiosity, might be biased in parental reporting. Clear counterfactuals in our study depend on the absence of relative parental bias between children born with clubfoot and the nearest‐age sibling. If parents systematically and subjectively inflate (deflate) outcomes for *untreated* children born with clubfoot relative to nearest‐age siblings it would result in a downward (upward) bias in our estimates of congenital clubfoot status. If parents systematically and subjectively inflate (deflate) outcomes for *treated* children born with clubfoot relative to nearest‐age siblings it would result in an upward (downward) bias in our estimates of congenital clubfoot status. However, if parents exhibit a uniform upward or downward bias in their evaluation that is consistent across children, it would not produce a bias in our estimations because this bias would be absorbed in the household‐level fixed effect.

### 3.7 Heterogeneous Effects

Estimations in Supporting Information S1: Appendix Table A12 provide little evidence of heterogeneous treatment effects by gender for either clubfoot status or treatment. Clubfoot is approximately twice as common among male children, which aligns with our sample. Our estimates suggest that boys are slightly less affected by congenital clubfoot status, though the coefficient is strongly significant only for social outcomes and marginally significant for the overall IHD index. Early treatment also appears to be more important for boys. Additionally, the table shows no systematic evidence of heterogeneous treatment effects by income level.^5^

### 3.8 Barriers to Treatment

Given the tragic declines across key life outcomes from untreated clubfoot and the effectiveness of early treatment, one may wonder why such a large portion of children born with clubfoot remain untreated. In LMICs, numerous barriers hinder individuals from seeking essential health treatments, exacerbating the burden of both disease and congenital conditions. The global health literature has highlighted a series of prominent obstacles: financial constraints, geographical frictions, perceptions of sub‐standard care, cultural beliefs, and social stigmas surrounding certain illnesses.

Drew et al. (2016) use a socio‐ecological model to study five interrelated factors that affect patient access and engagement with clubfoot treatment in LMICs: intrapersonal, interpersonal, institutional, socio‐cultural, and public policy barriers. They find the most binding factors to be intrapersonal, institutional, and public policy barriers. Intrapersonal barriers included a lack of cash needed for treatment and the additional responsibilities associated with clubfoot care. Institutional barriers include long distances to treatment centers and insufficient information about treatments, and the challenges of maintaining home treatment. At the public policy level, the two‐tiered healthcare system often present in LMICs, such as Ethiopia, have made it impossible for some groups to access timely care (Drew et al. 2016).

As part of our fieldwork, we investigated which obstacles to clubfoot treatment, across these different areas, carried the most weight within our Ethiopian sample. Informed by the literature and guided by the insight of our NGO partner, we developed a series of questions on barriers to clubfoot treatment that we administered to a subset of our sample (*N* = 136). Responses were collected on a total of 14 questions relating to distinct factors that could prevent seeking treatment. These factors were: (1) cost, (2) distance, (3) knowledge that condition is treatable, (4) knowledge about the severity of the condition, (5) knowledge of availability of treatment, (6) time, (7) social pressure, (8) belief that disability is part of a Divine plan, (9) trust in traditional healers, (10) feelings of shame associated with treatment, (11) fear of ineffective treatment, (12) false belief that the condition had been treated, (13) worry about quality of clinics, and (14) long wait times.

Table 3 shows that of the 14 barriers we included in our survey, there were six that the majority of mothers listed to be a strong deterrent to seeking treatment: lack of knowledge about the availability of treatment (76.47%); living too far away from available clubfoot treatment centers (70.59%); lack of time for treatment due to other obligations (66.91%); inability to afford costs associated with treatment (64.71%); lack of knowledge that condition is treatable (63.97%); and lack of knowledge about severity of the condition (56.62%).

**TABLE 3:** Barriers to clubfoot treatment.

No.	Barrier	Prevalence (%)	*N*
1	Treatment availability	76.47	136
2	Distance	70.59	136
3	Time	66.91	136
4	Cost	64.71	136
5	Doubt clubfoot treatable	63.97	136
6	Unclear clubfoot severity	56.61	136
7	Shame	36.76	136
8	Treatment ineffective	34.56	136
9	Wait time	28.67	136
10	Pressure	20.59	136
11	Assumed treated	19.12	136
12	Belief availability	18.38	136
13	Traditional healers	7.35	136
14	Clinic quality	5.15	136

*Note:* Respondents were allowed to list as many barriers to treatment as applicable in their context.

The responses from our sample point to logistical and informational barriers as a greater obstacle for accessing clubfoot treatment relative to socio‐cultural factors. At least in our Ethiopian context, this suggests a largely informational and supply‐side challenge, as opposed to constraints deeply rooted in cultural beliefs or social norms. These responses also portend well for the impact of additional resources allocated to increasing both information and access, where high impact is likely to be matched by high take‐up, making impact at an “intent‐to‐treat” (ITT) level likely to be high. Moreover, it provides a rationale for development NGOs and specialized government ministries seeking to make treatment of congenital birth defects such as clubfoot available in remote regions. By addressing tangible hurdles such as the dissemination of information about treatment options and access to treatment facilities, the volume of treatment of a substantively life‐changing intervention might be significantly increased.

## 4 Discussion

Addressing issues of inequity and inclusion for people coping with disabilities in LMICs is a theme throughout the U.N.’s Sustainable Development Goals. However, there is a lack of research documenting the causal impacts of congenital impairments and the impacts of treatment across human life outcomes. Our paper helps to close this evidence gap by establishing valid counterfactuals on life outcomes for congenital clubfoot status and for the Ponseti clubfoot treatment. We summarize here our main conclusions.

First, we estimate that untreated congenital clubfoot causes an enormous 1.3219σ decline in a holistic human flourishing index, mediated by large and statistically significant declines in physical mobility, mental health, social inclusion, educational, and faith outcomes. To provide a sense of this magnitude, the negative impacts we estimate from untreated clubfoot are 3.6× times larger than those found from untreated cleft palate which, in a study of the work of Operation Smile in India, saw a loss in a similar index of human flourishing of 0.37σ (Wydick et al. 2022).

Second, we find the Ponseti clubfoot intervention to realize large and significant impacts across all of our five facets of human flourishing. Overall, we estimate that early treatment restores between 71% and 82% of human flourishing lost from congenital clubfoot in our Ethiopian sample depending on the type of summary index. Hope Walks lists its cost of the intervention at approximately US$500 per patient in the low‐income countries where it operates. We find impacts on human flourishing from clubfoot surgery to compare favorably to those from cleft palate repair (Wydick et al. 2022), which carries a similar cost to health NGOs in low‐income countries. Cataract surgery has also been shown to have far‐ranging impacts across many life outcomes, including employment and wages (Flessa 2022). The estimated cost of cataract surgery (and follow‐up) in LMICs ranges between US$300–400 (Meltzer et al. 2017; Flessa 2022), which is slightly lower than for clubfoot intervention. However, it is difficult to make a direct comparison of impacts on human flourishing outcomes with the cataract research, which does not use similar causal methods and studies a different set of categorical outcomes.^6^

Interventions that realize large, clear, and broad effects on human outcomes are uncommon, and we believe the reason for these unusually large effects lies in a combination of factors. One is that the Ponseti method is a medically proven treatment, used throughout western medicine as an extremely effective intervention to such an extent that world‐class athletes born with clubfoot have excelled at the highest levels of athletic achievement after receiving the Ponseti treatment.^7^ However, as our data illustrate, the alternative to proper clubfoot treatment in a low‐income country such as Ethiopia is tragically bleak. Indeed, one could argue that individuals with untreated congenital conditions from impoverished households in low‐income countries may be among the most disadvantaged persons on a global level. This dire counterfactual, combined with a very effective medical intervention, even when applied imperfectly, creates the scope for extremely large treatment effects. Moreover, the intervention generates broad spillover effects across key developmental outcomes, further amplifying its impact.

Importantly, our results on the impacts of clubfoot treatment on facets of human flourishing find early treatment (≤ 6 months) to be essential for significant impact on later‐life outcomes. Indeed, we find no evidence of life outcome impacts for clubfoot intervention that is commenced after this point. A clear implication from this is the importance of informational campaigns in rural and remote areas that identify children born with clubfoot so that they can be treated in early infancy.

## Ethics Statement

This research was reviewed and approved by the Institutional Review Board for the Protection of Human Subjects under IRB Protocol #1623 at the University of San Francisco.

## Conflicts of Interest

The authors declare no conflicts of interest.

## Supporting information


Supporting Information S1

## Data Availability

The data and material collected for this research are available upon request.

## Endnotes

^1^
Our data does not delineate whether a child's religious faith community is Muslim, Orthodox Christian, or Protestant Christian, the predominant religious groups in Ethiopia.

^2^
There are two categories analyzed in the Pirani Scale, the midfoot and the hindfoot, with three sections in each category. Scores range from 1 to 6, with larger numbers indicating higher severity of deformities (Dryer and Davis 2006).

^3^
Recent studies analyze the diverse national policy and support frameworks for young carers, many of whom are siblings taking on caregiving responsibilities within their families (Chevrier et al. 2022; Leu et al. 2022).

^4^
Supporting Information S1: Appendix Table A10 shows all of the faith sub‐areas to be significantly and negatively impacted, with simple participation in the faith community showing the largest negative impacts.

^5^
18 of our estimated coefficients interacted with income are of relatively low magnitude and none reach statistical significance.

^6^
Beyond surgical interventions, deworming programs targeting children have been celebrated as a benchmark health intervention for cost‐effectiveness, with estimates as low as $0.50 per child treated [WHO]. Hotez et al. (2009) found that mass deworming interventions in children led to significant improvements in cognitive function and school attendance. Hamory et al. (2021) find significant impacts from early deworming on key life outcomes 20 years after initial intervention. Deworming programs, however, often require large campaigns, high levels of compliance, and sustained intervention.

^7^
Olympic figure‐skating champion Kristi Yamaguchi, football quarterback Troy Aikman, and U.S. soccer player Mia Hamm were each born with congenital clubfoot.
