# Supplementary material for: Hope Walks: The Impact of Clubfoot Treatment on Human Flourishing in Ethiopia
Source: Health Econ. 2025 Sep 18;35(1):25–35. doi: 10.1002/hec.70040 (PMC12680910; doi:10.1002/hec.70040)
Supplement: Supplementary file 1 — Supporting Information S1 [file HEC-35-25-s001.pdf]

Hope Walks: The Impact of Clubfoot Treatment on Human Flourishing in Ethiopia

Bruce Wydick[1,2,3], Gianna Camacho,[4] and Patrizio Piraino,[2]

September 11, 2025

Keywords: Clubfoot, Human Flourishing, Reparative Surgeries, Program Evaluation

Abstract

Children born with severe congenital anomalies in low-income countries rank among the most disadvantaged among the global ultra-poor. We study the impact of the clubfoot disability and its treatment across multiple dimensions of human flourishing on data collected from 564 children in Ethiopia. Working with Hope Walks, an organization that funds clubfoot interventions in numerous countries, we use a quasi difference-in-differences approach that generates counterfactual outcomes from the nearest-age siblings of children born with clubfoot, nested within a family-level fixed effect. We find that clubfoot status (early treatment) results in a disability (restoration) of -1.44σ (0.91σ) in physical mobility, -1.17σ (0.79σ) in mental health, -1.07σ (0.64σ) in social inclusion, -0.48σ (0.98σ) in an education index, -0.76σ (0.42σ) in religious faith, and -1.19σ (0.79σ) in an aggregate index of human flourishing (all $p < 0.05$). We attribute the large, broad, and significant impacts from clubfoot treatment to (i) a highly effective medical intervention that is (ii) carried out in an impoverished setting with scarce existing support for children born with disabilities, which (iii) broadly generates spillover effects across key development outcomes.

Affiliations: [1]University of San Francisco, [2]University of Notre Dame, Kellogg Institute for International Studies and CEIDS; [3]Center for Effective Global Action (CEGA), University of California at Berkeley; [4] Department of Public Health, Commonwealth of the Northern Mariana Islands. Corresponding author, Email: wydick@usfca.edu, Department of Economics, University of San Francisco, San Francisco, CA 94117.
Funding: This work was supported by the Kellogg Institute for International Studies and the Ford Family Program at the University of Notre Dame, the University of San Francisco, and an anonymous donor.
Acknowledgments: We wish to thank Scott Reichenbach, Jai Sarma, and Endashawu Abera for their partnership with this project and helpful comments and input by Chris Ahlin, Alessandra Cassar, Andrew Hobbs, Kira Villa, Paul Niehaus, and attendees of the CEIDS online seminar and 2023 and 2024 workshops. We are grateful to the Kellogg Institute and Ford Family Program at Notre Dame, the University of San Francisco, and an anonymous donor for research funding. All errors are our own. Data is available upon request.

Online Appendix

In the appendix we provide a more detailed analysis of the outcomes in each of our specific areas of human flourishing with accompanying figures and tables.

Physical Outcomes

Figure A1 shows a kernel density function showing clubfoot and treatment status related to physical mobility. The figure shows siblings of children born with clubfoot (who themselves were not born with clubfoot) to clearly have the highest level of mobility, followed by children with early treated clubfoot, children with late treated clubfoot, and children born with clubfoot who remained untreated at the time of the survey. Early treatment appears to function particularly well at reducing the left tail of the distribution, though density at the peak levels of mobility is lower than children born without clubfoot.

Table A1 references results from foot appearance and size. Children born with clubfoot score 0.73σ lower on a scale relating to being able to fit into shoes of their choice. Mothers are much less likely to report that their child's feet are the same size (column 2), that they appear to others as being of a different size (column 3), that their feet appear "normal" to others (column 4). An index of foot appearance lies 1.5σ below children without clubfoot. Early treatment for clubfoot does not erase perceptions of foot disfigurement or size, but significantly mitigates these problems, particularly in the perceptions that feet appear "normal" to others. We find no significant impact from late treated clubfoot (initiation of treatment after six months of age) on foot appearance. In our estimations, early Ponseti treatment restores 52.1% of our index of foot appearance, and a joint F-test of the three coefficients (born with clubfoot, treatment, and early treatment) rejects full restoration at $p < 0.01$, although it is clear that the intervention is effective at mitigating about half the magnitude of problems related to foot-appearance resulting from clubfoot.[1]

Children born with clubfoot in the Ethiopian sample unsurprisingly report 1.09σ lower on the commonly used mobility scale.[2] As can be seen in Table A2, congenital clubfoot reduces every facet of mobility in our survey by over a standard deviation: mobility scale, distance able to walk, comfort walking, ability to play sports, enjoyment of sports, and complaints of tiredness of feet and legs when active. Late-treated clubfoot has no significant impact on later mobility, and while early treatment does not restore full mobility

[1]An index of foot pain, not shown in the table, increases by 1.35σ and is reduced by early treatment by 0.34σ.

[2]The mobility scale grades on a scale of 1-use of wheelchair only; 2-use of walker; 3-use of crutches; 4-use of sticks; 5-independent on level surfaces to 6-independent mobility on all surfaces.

(p ¡ 0.01) on average it restores 50.1% of mobility based on our mobility index.

Psychological Outcomes

Figure A2 shows the density function for mental health of children treated early for clubfoot to nearly replicate the density across mental health outcomes of their siblings, while late-treated and untreated children display density functions that lie increasingly further to the left, respectively. Table A3 shows the great toll to which a disability such as clubfoot inflicts on the mental health of children in a low-income country. Based on Likert scale questions, mothers indicate that when children born with clubfoot are mistreated, they are far more likely to believe that they deserve the mistreatment. Prevalence of worrying (1.06σ) and self-criticism (1.51σ) are more than a standard deviation higher, feeling respected (-1.15σ) and comfortable with their appearance (-1.64σ) more than a standard deviation lower with an index of self-esteem -1.53σ lower than nearest-age siblings. Late treated clubfoot shows no significantly positive impact on self-esteem except perhaps in comfort with one's appearance, while early treatment has large effects on restoring self-esteem (66.4%) although not fully ($p < 0.01$).

Impacts of congenital clubfoot and clubfoot treatment on hope and aspirations are in Table A4. These relate to believing that they will finish secondary school, obtain a university degree, have a ”good job” in the future, be ”happily married”, and ”have dreams and plans for a good life”. They indicate that the effects of both clubfoot status and treatment are somewhat lower on children's hope and aspirations than on self esteem and show impacts in the -0.3σ to -0.7σ range, but that early treatment is able to partially restore (p ¡ 0.01) most of these hopes and aspirations by about 0.3σ to 0.4σ standard deviations, overall 62.4% of the damage to these areas caused by clubfoot.

Table A5 gives results for impacts on anxiety, specifically for Likert scale responses related to a child's general nervousness, anxiety about specific life events, or fear of an unknown tragic event. Negative impacts from congenital clubfoot on the prevalence of anxiety are smaller than the effects on self-esteem, but greater than the effects on hope and aspirations, lying in the 0.7σ to 0.9σ range with the impact on the aggregated anxiety index at 0.83σ. Children with late-treated clubfoot actually seem to have *higher* rates of anxiety than untreated children, but the impacts from early treatment are such that they are able to mitigate 40.7% of clubfoot-induced anxiety.

Along with lowering self-esteem, hope, and aspirations and raising levels of anxiety, we find that clubfoot also gives rise to depression. Table A6 shows our index of depression, which consists of children having low motivation, feeling ”down, depressed or hopeless”,

feeling ”bad about themselves”, and feeling unhappy. Depression increases by 1.04σ with congenital clubfoot, but decreases by 0.79σ with early treatment, although later treatment is associate with slightly higher (though mostly statistically insignificant) increases in depression. Overall, our estimates show early treatment is able to mitigate 69.32% of this depression. Our overall psychological health index (column 7 in Table A6) decreases by 1.17σ from clubfoot, where our estimates show early treatment is able to restore 57.3% of the decline in mental health resulting from clubfoot status though we can reject full restoration at $p < 0.01$.

Social Outcomes

The first row of Table A7 shows the impact on various facets of children's social inclusion: hours per day playing outside the home, number of friends, leaving the house in the company of friends, the extent to which a child feels included in society, quality of a child's relationships, frequency of bullying and teasing, and the extent to which the extended family are ”proud and happy to have the child as a family member”. There is no significant effect of clubfoot on the number of friends a child has, but there are large effects on any measure of social inclusion that requires mobility outside the home. Negative effects on social inclusion display a wide range, from insignificant (friendships) to 1.4σ (frequency of bullying), where the impact on the overall social inclusion index is -1.15σ.

We estimate that early clubfoot treatment restores 56.5% of the loss of social inclusion due to congenital clubfoot, where the impact on the reduction in bullying is especially notable. Even late treatment reduces approximately half (49.6%) of bullying from clubfoot, but early treatment reduces 86.7% of bullying, obviously creating an immense positive impact on the lives of children in one of the areas most negatively affected by clubfoot status. Figure A3 shows the marked difference in clubfoot treatment status, where raw outcomes from early treatment are virtually identical to nearest-age siblings and late treated and untreated children show lower modes and much thicker left tails.

Table A8 shows results on pro-social behavior, where congenital clubfoot causes problems with children meeting new people (-0.53σ), choosing to remain alone (0.98σ), sharing food, toys and other things with others (-0.40σ), feeling nervous around new people (-1.08σ). Clubfoot status has no significant effect on a child being helpful to others when someone else is feeling upset or ill. In areas of pro-social behavior where there are negative impacts from clubfoot, late treatment is statistically ineffective, but early treatment on average restores 57.2% of pro-sociality.

Educational Outcomes

Figure A4 shows kernel density outcomes over an educational outcomes index. Impacts from both clubfoot and clubfoot treatment on education are lower than physical, psychological, and social outcomes, but are significantly manifest in the data. Modes in educational outcomes in the figure are relatively similar, but many late-treated and untreated children born with clubfoot display a far higher prevalence of outcomes that are 2-3σ below the mean.

Impact results are shown in Table A9 and show significantly lower attendance at pre-kinder schools and kindergarten, which typically occurs before primary school begins at age 7 in Ethiopia. Children born with clubfoot are less likely to be enrolled in school, slightly more likely to be enrolled at a lower level in school than others their age, and have lower levels of school performance, although the latter two outcomes do not reach statistical significance. An education index of these outcomes shows an impact of -0.48σ on education outcomes ($p < 0.01$).

Impacts on educational outcomes from early treatment are substantial, more than fully restoring age-appropriate status for school level such that early clubfoot status restores 117% of outcomes in our schooling index. It is possible that this may be due to a combination of factors.

While early treatment significantly restores mobility, it only partially restores it generally (see Table A2), but fully restores it with respect to enrollment in school. This may create a scenario for some children in which their ability to attend school combined with their remaining (somewhat) lower ability to play sports leads them to spend more time in study, thus accounting for the very substantial (0.56σ) impact (see column 4) on school performance from early treatment.

Faith and Spirituality Outcomes

Figure A5 shows kernel densities across clubfoot status for faith and spirituality outcomes, where outcomes show higher levels of spiritual belief and engagement in religious activities for children born without clubfoot and early-treated children relative to late treated and untreated children.

Table A10 shows that these results appear to be driven at least partially by the ability of children to be physically present at religious gatherings, where there are large impacts from early treatment on faith community involvement and participation in youth activities. We find that clubfoot treatment, regardless of timing, restores spiritual belief: Clubfoot status results in a difference in a 0-10 degree index to the question "How important is

religious faith and the local faith community in this child's life?" of -0.54σ. Treatment for clubfoot restores this degree of importance of religious faith by a nearly identical 0.53σ, where the timing of treatment (early or late) yields no significant difference to impact.

Figure A1: Physical Mobility Across Clubfoot Treatment Status

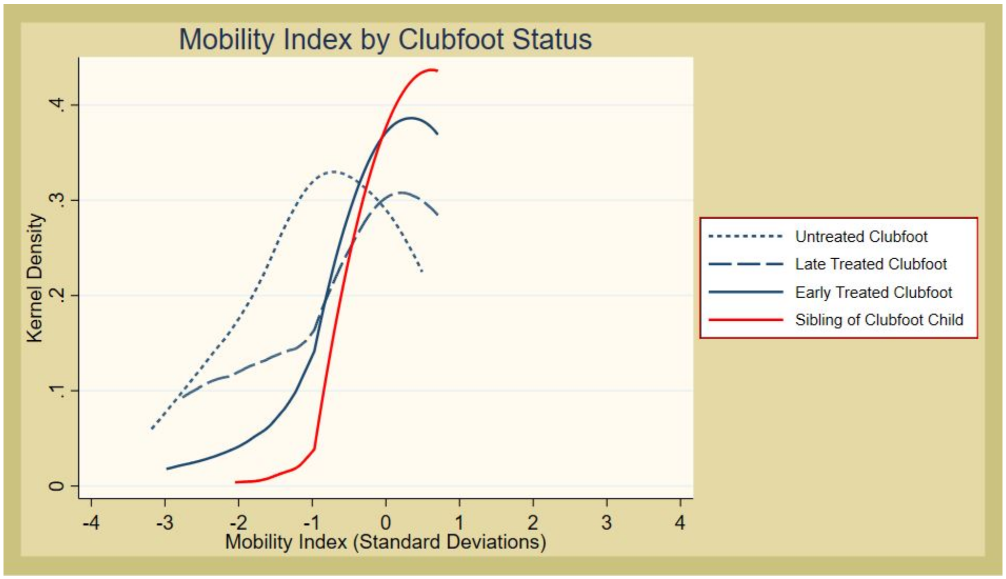

Figure A2: Psychological Health Index Across Clubfoot Treatment Status

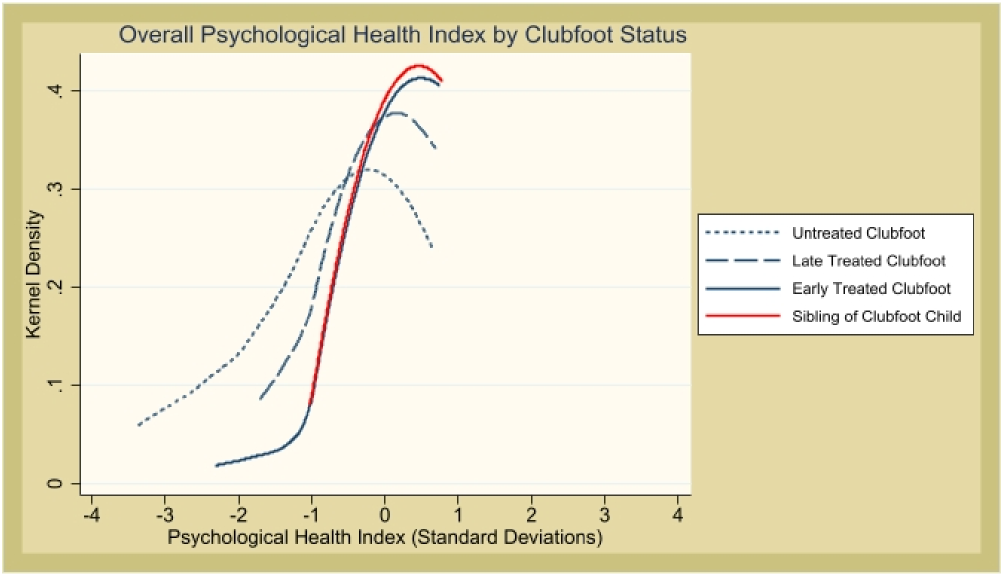

Figure A3: Social Inclusion Index Across Clubfoot Treatment Status

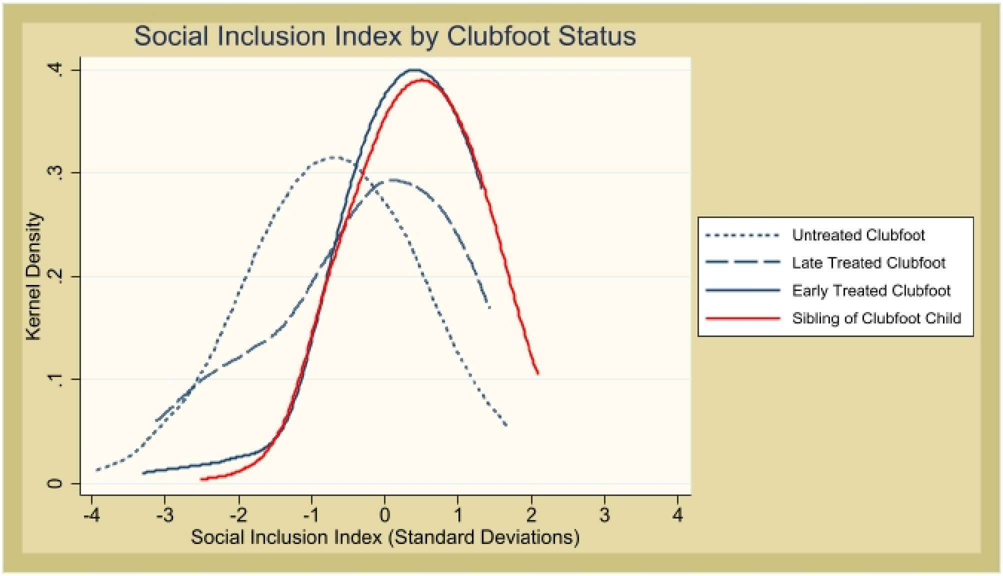

Figure A4: Education Index Across Clubfoot Treatment Status

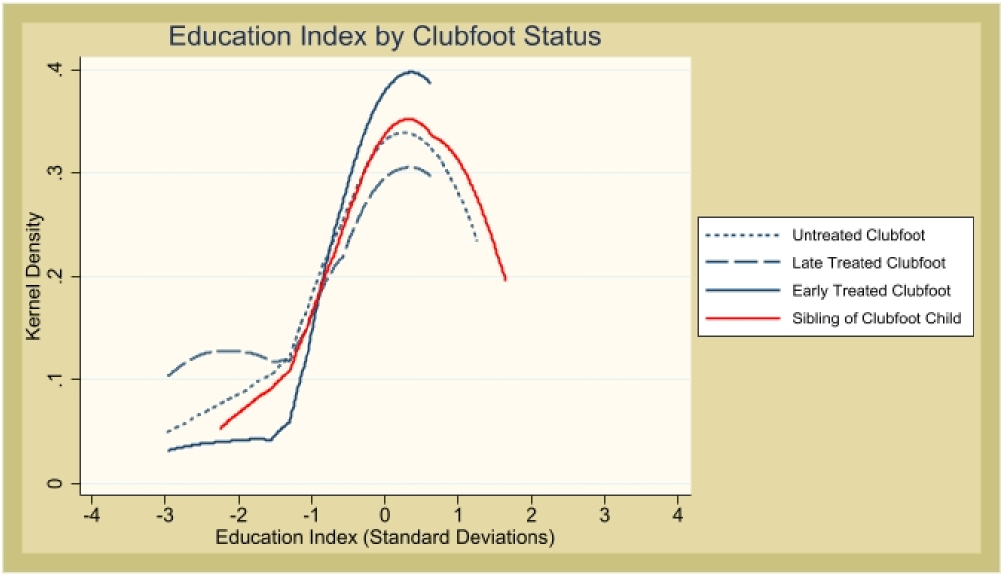

Figure A5: Faith and Spirituality Index Across Clubfoot Treatment Status

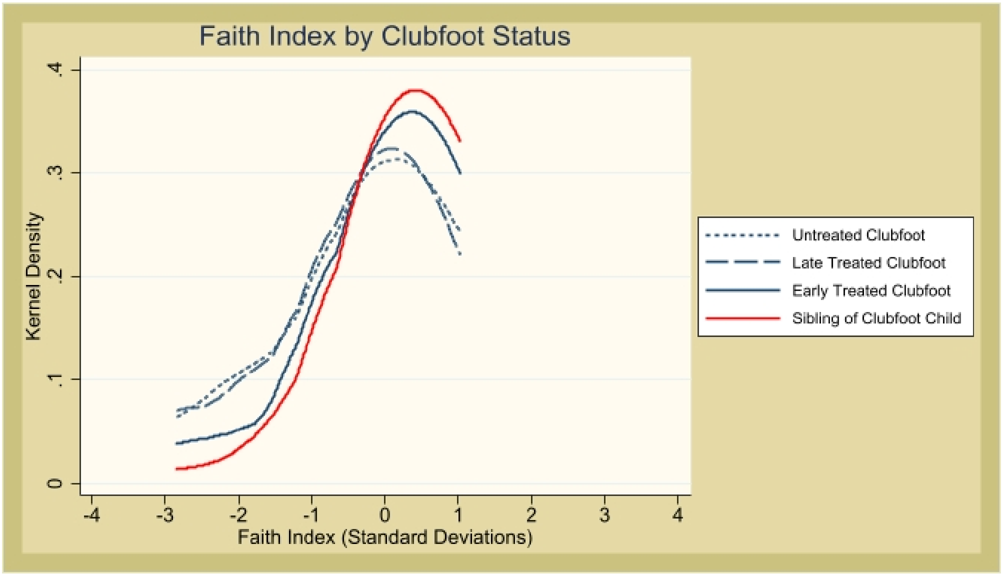

Table A1: Foot Symmetry and Appearance

	(1) Shoes Fit	(2) Feet Symm	(3) Appearance	(4) Perception	(5) Foot Index
Born with Clubfoot	-0.726***	-0.605***	-1.672***	-1.746***	-1.479***
	(0.129)	(0.124)	(0.0924)	(0.0843)	(0.0955)
Treated for Clubfoot	-0.537*	-0.384	0.256	0.184	-0.150
	(0.259)	(0.247)	(0.185)	(0.172)	(0.209)
Early Treatment	0.926***	0.476*	0.745***	0.798***	0.917***
	(0.234)	(0.227)	(0.192)	(0.183)	(0.206)
N	564	564	564	564	564
MotherFE	Yes	Yes	Yes	Yes	Yes

Clustered standard errors at the household level are in parentheses.

Regressions control for age, gender, and birth order of children.

Percent restoration of index with early surgery = 52.1%

Joint (Index) test of Clubfoot + Treatment + Early Treatment = -0.71, $p < 0.01$

* $p < 0.05$, ** $p < 0.01$, *** $p < 0.001$

Table A2: Mobility and Participation in Sports Activities

	(1) Mobility	(2) WalkDis	(3) WalkCom	(4) SptAbl	(5) SptEnj	(6) Tired	(7) Mob. Index
Born Clubfoot	-1.086***	-1.254***	-1.012***	-1.432***	-1.546***	1.120***	-1.443***
	(0.110)	(0.112)	(0.115)	(0.101)	(0.0991)	(0.110)	(0.0893)
Treated Clubfoot	-0.388	-0.212	-0.521*	-0.0825	0.00835	-0.224	-0.188
	(0.232)	(0.213)	(0.219)	(0.190)	(0.187)	(0.218)	(0.196)
Early Treat	0.836**	0.821***	1.046***	0.818***	0.879***	-0.271	0.905***
	(0.253)	(0.216)	(0.221)	(0.199)	(0.198)	(0.207)	(0.212)
N	564	564	564	564	564	564	564
MotherFE	YES	YES	YES	YES	YES	YES	YES

Standard errors clustered at the household level in parentheses.
Regressions control for age, gender, and birth order of children.
Percent restoration of index with early surgery = 50.1%
Joint (Index) test of Clubfoot + Treatment + Early Treatment, -0.72, $p < 0.01$
* $p < 0.05$, ** $p < 0.01$, *** $p < 0.001$

Table A3: Self-Esteem

	(1) Mistreats	(2) Worries	(3) Respected	(4) Appearance	(5) Selfcritic	(6) Self-Est. Index
Born Clubfoot	0.937***	1.058***	-1.157***	-1.638***	1.515***	-1.526***
	(0.130)	(0.139)	(0.115)	(0.102)	(0.112)	(0.0946)
Treated Clubfoot	-0.108	-0.309	0.154	0.452*	-0.638***	0.402*
	(0.201)	(0.206)	(0.182)	(0.180)	(0.187)	(0.162)
Early Treat	-0.518**	-0.409*	0.604***	0.551**	-0.432*	0.608***
	(0.183)	(0.181)	(0.159)	(0.182)	(0.179)	(0.165)
N	564	564	564	564	564	564
MotherFE	Yes	Yes	Yes	Yes	Yes	Yes

Standard errors clustered at the household level in parentheses.
Regressions control for age, gender, and birth order of children.
Percent restoration of index with early surgery = 66.4%
Joint (Index) test of Clubfoot + Treatment + Early Treatment, -0.52, $p < 0.01$
* $p < 0.05$), ** $p < 0.01$, *** $p < 0.001$

Table A4: Hope and Aspirations

	(1) SecondSch	(2) University	(3) GoodJob	(4) HapMarried	(5) Dreams	(6) Aspir Index
Born Clubfoot	-0.668***	-0.746***	-0.271*	-0.625***	-0.695***	-0.785***
	(0.107)	(0.111)	(0.123)	(0.0949)	(0.100)	(0.108)
Treated Clubfoot	0.0919	-0.0886	0.212	-0.0164	0.0224	0.0578
	(0.173)	(0.181)	(0.227)	(0.149)	(0.167)	(0.176)
Early Treat	0.334*	0.516***	-0.0392	0.414***	0.412**	0.428**
	(0.137)	(0.148)	(0.213)	(0.123)	(0.134)	(0.150)
N	564	564	564	564	564	564
MotherFE	Yes	Yes	Yes	Yes	Yes	Yes

Standard errors clustered at the household level in parentheses.
Regressions control for age, gender, and birth order of children.
Percent restoration of index with early surgery = 62.4%
Joint (Index) test of Clubfoot + Treatment + Early Treatment, -0.30, $p < 0.01$
* $p < 0.05$),** $p < 0.01$, *** $p < 0.001$

Table A5: Anxiety

	(1) Nervous	(2) Anxiety	(3) Fear	(4) Anxiety Index
Born Clubfoot	0.748***	0.875***	0.820***	0.835***
	(0.122)	(0.123)	(0.122)	(0.123)
Treated Clubfoot	0.679**	0.653*	0.602*	0.661**
	(0.250)	(0.255)	(0.252)	(0.252)
Early Treat	-0.939***	-1.049***	-0.938***	-1.000***
	(0.216)	(0.223)	(0.217)	(0.220)
N	564	564	564	564
MotherFE	Yes	Yes	Yes	Yes

Standard errors clustered at the household level in parentheses.
Regressions control for age, gender, and birth order of children.
Percent restoration of index with early surgery = 40.7%
Joint (Index) test of Clubfoot + Treatment + Early Treatment, -0.50, $p < 0.01$.
* $p < 0.05$, ** $p < 0.01$, *** $p < 0.001$

Table A6: Depression

	(1) Low Motiv	(2) Depressed	(3) FeelFailure	(4) Happy	(5) Depr Index	(6) Psych Index
Born Clubfoot	0.408***	0.870***	0.911***	-1.375***	1.042***	-1.171***
	(0.0618)	(0.122)	(0.137)	(0.0996)	(0.101)	(0.0922)
Treated Clubfoot	0.0249	0.598*	0.0469	-0.0778	0.218	-0.117
	(0.127)	(0.255)	(0.241)	(0.194)	(0.193)	(0.176)
Early Treat	-0.280**	-0.992***	-0.637***	0.797***	-0.791***	0.790***
	(0.104)	(0.219)	(0.181)	(0.184)	(0.175)	(0.167)
N	564	564	564	564	564	564
MotherFE	Yes	Yes	Yes	Yes	Yes	Yes

Standard errors clustered at the household level in parentheses.
Regressions control for age, gender, and birth order of children.
Percent mitigation of depression with early surgery = 69.2%
Percent restoration of psychological health index with early surgery = 57.3%
Joint (Index) test of Clubfoot + Treatment + Early Treatment, -0.50, $p < 0.01$.
* $p < 0.05$, ** $p < 0.01$, *** $p < 0.001$

Table A7: Social Inclusion

	(1) Play	(2) Friends	(3) Outside	(4) Incl'd	(5) Relat	(6) Bullied	(7) FProud	(8) SocInc Index
Bn CF	-0.630***	0.0915	-0.453***	-0.983***	-1.085***	1.426***	-0.278*	-1.152***
	(0.120)	(0.132)	(0.0945)	(0.115)	(0.0980)	(0.121)	(0.108)	(0.0957)
T'ed CF	-0.512*	-0.267	0.100	-0.314	-0.226	-0.709***	0.0136	-0.143
	(0.215)	(0.220)	(0.171)	(0.205)	(0.187)	(0.186)	(0.156)	(0.188)
Early Trt	0.822***	0.0593	-0.133	0.854***	0.916***	-0.527**	0.178	0.785***
	(0.243)	(0.218)	(0.203)	(0.197)	(0.184)	(0.159)	(0.150)	(0.200)
N	564	564	564	564	564	564	564	564
MotherFE	Yes	Yes	Yes	Yes	Yes	Yes	Yes	Yes

Standard errors clustered at the household level in parentheses.
Regressions control for age, gender, and birth order of children.
Percent restoration of index with early surgery = 56.5%
Joint (Index) test of Clubfoot + Treatment + Early Treatment, -0.51, $p < 0.01$.
* $p < 0.05$, ** $p < 0.01$, *** $p < 0.001$

Table A8: Pro-Social Behavior

	(1) MeetPeople	(2) ChooseAlone	(3) Helpful	(4) Shares	(5) NervMtg	(6) SocBehav Index
Born Clubfoot	-0.525***	0.977***	-0.100	-0.402*	1.077***	-0.826***
	(0.0883)	(0.122)	(0.109)	(0.156)	(0.120)	(0.0955)
Treated Clubfoot	0.0575	-0.0292	0.249	0.132	0.187	0.0752
	(0.134)	(0.193)	(0.183)	(0.223)	(0.203)	(0.151)
Early Treat	0.259*	-0.449**	-0.168	0.195	-0.756***	0.400**
	(0.120)	(0.169)	(0.152)	(0.179)	(0.193)	(0.148)
N	564	564	564	564	564	564
MotherFE	Yes	Yes	Yes	Yes	Yes	Yes

Standard errors clustered at the household level in parentheses.

Regressions control for age, gender, and birth order of children.

Percent restoration of index with early surgery = 57.2%

Joint (Index) test of Clubfoot + Treatment + Early Treatment, -0.35, $p < 0.01$.

* $p < 0.05$, ** $p < 0.01$, *** $p < 0.001$

Table A9: Education

	(1) Pre-Kinder	(2) Current School	(3) School Level	(4) School Perf	(5) Education Index
Born Clubfoot	-0.194***	-0.189***	-0.111	-0.182	-0.477***
	(0.0394)	(0.0382)	(0.124)	(0.115)	(0.116)
Treated Clubfoot	-0.162*	-0.140	-0.0626	-0.297	-0.420
	(0.0794)	(0.0771)	(0.204)	(0.223)	(0.238)
Early Treat	0.264***	0.216**	0.569*	0.902***	0.980***
	(0.0773)	(0.0793)	(0.220)	(0.238)	(0.249)
N	564	564	564	564	564
MotherFE	Yes	Yes	Yes	Yes	Yes

Standard errors clustered at the household level in parentheses.

Regressions control for age, gender, and birth order of children.

Percent restoration of index with early surgery = 117.4%

Joint (Index) test of Clubfoot + Treatment + Early Treatment, 0.08, $p = 0.67$.

* $p < 0.05$, ** $p < 0.01$, *** $p < 0.001$

Table A10: Faith and Spirituality

	(1)	(2)	(3)	(4)
	Faith Community	Faith Important	Youth Activities	Faith Index
Born Clubfoot	-0.880***	-0.544***	-0.528***	-0.762***
	(0.105)	(0.113)	(0.0759)	(0.0942)
Treated Clubfoot	-0.431*	0.536*	-0.290	-0.0721
	(0.196)	(0.216)	(0.151)	(0.156)
Early Treat	0.776***	-0.0428	0.329*	0.415*
	(0.196)	(0.196)	(0.163)	(0.160)
N	564	564	564	564
MotherFE	Yes	Yes	Yes	Yes

Standard errors clustered at the household level in parentheses.

Regressions control for age, gender, and birth order of children.

Percent restoration of index with early surgery = 44.7%

Joint (Index) test of Clubfoot + Treatment + Early Treatment, -0.42, $p < 0.01$.

* $p < 0.05$, ** $p < 0.01$, *** $p < 0.001$

Table A11: Summary Outcomes: Human Flourishing using Anderson Index

	(1)	(2)	(3)	(4)	(5)	(6)
	Physical	Psych	Social	Educ	Faith	HF Index
Born Clubfoot	-1.326***	-1.208***	-0.760***	-0.519***	-0.702***	-1.067***
	(0.0924)	(0.0917)	(0.0961)	(0.119)	(0.0928)	(0.0976)
Treated Clubfoot	-0.199	-0.0745	-0.00500	-0.385	0.0312	-0.218
	(0.202)	(0.180)	(0.179)	(0.231)	(0.151)	(0.192)
Early Treat	0.811***	0.779***	0.405*	0.917***	0.297*	0.888***
	(0.218)	(0.169)	(0.191)	(0.244)	(0.154)	(0.202)
N	564	564	564	564	564	564
MotherFE	Yes	Yes	Yes	Yes	Yes	Yes

Standard errors in parentheses.

Regressions control for age, gender, and birth order of children.

Joint (Index) Test of Clubfoot + Treatment + Early Treatment, -0.71, $p < 0.01$.

* $p < 0.05$, ** $p < 0.01$, *** $p < 0.001$

Table A12: Heterogeneous Treatment Effects by Gender and Poverty Level

	(1) Physical	(2) Psych	(3) Social	(4) Educ	(5) Faith	(6) IHD Index
Born Clubfoot	-1.602***	-1.429***	-1.566***	-0.590**	-0.849***	-1.584***
	(0.163)	(0.191)	(0.207)	(0.211)	(0.221)	(0.199)
Male_x_BornCF	0.278	0.233	0.621**	0.301	0.211	0.396*
	(0.185)	(0.216)	(0.229)	(0.224)	(0.265)	(0.255)
LowInc_x_BornCF	-0.114	-0.0495	0.179	-0.114	0.136	0.068
	(0.182)	(0.222)	(0.199)	(0.196)	(0.216)	(0.188)
Treated Clubfoot	0.198	0.0813	0.588	0.103	0.142	0.295
	(0.363)	(0.430)	(0.402)	(0.436)	(0.345)	(0.372)
Early Treat	0.722*	0.887*	0.611	0.635	0.377	0.897*
	(0.375)	(0.398)	(0.386)	(0.425)	(0.337)	(0.353)
Male_x_Treated	-0.925*	-0.861	-1.232**	-0.363	-0.707	-1.035*
	(0.401)	(0.446)	(0.420)	(0.445)	(0.387)	(0.405)
Male_x_EarlyTreat	0.675	0.667	0.729	0.0327	0.478	0.665
	(0.418)	(0.407)	(0.410)	(0.445)	(0.388)	(0.398)
LowInc_x_Treated	0.300	0.470	-0.156	-0.529	-0.326	-0.141
	(0.407)	(0.448)	(0.414)	(0.428)	(0.390)	(0.404)
LowInc_x_EarlyTreat	-0.416	-0.425	0.0861	0.297	0.259	0.040
	(0.446)	(0.418)	(0.424)	(0.447)	(0.408)	(0.418)
N	564	564	564	564	564	564
MotherFE	No	No	No	No	No	No

Standard errors in parentheses

Regressions control for household income and age, gender, birth order of children.

* $p < 0.05$, ** $p < 0.01$, *** $p < 0.001$
